# Neurofilament Light Chain in Spinal Fluid and Plasma in Multiple System Atrophy – A Prospective, Longitudinal Biomarker Study

**DOI:** 10.21203/rs.3.rs-3201386/v1

**Published:** 2023-08-01

**Authors:** Wolfgang Singer, Ann M. Schmeichel, David M. Sletten, Tonette L. Gehrking, Jade A. Gehrking, Jorge Trejo-Lopez, Mariana D. Suarez, Jennifer K. Anderson, Pamela H. Bass, Timothy G. Lesnick, Phillip A. Low

**Affiliations:** 1 Department of Neurology, Mayo Clinic, Rochester, MN, USA; 2 Department of Laboratory Medicine and Pathology, Mayo Clinic, Rochester, MN, USA; 3 Department of Quantitative Health Sciences, Mayo Clinic, Rochester, MN, USA

## Abstract

**Purpose:**

There is a critical need for reliable diagnostic biomarkers as well as surrogate markers of disease progression in multiple system atrophy (MSA). Neurofilament light chain (NfL) has been reported to potentially meet those needs. We therefore sought to explore the value of NfL in plasma (NfL-p) in contrast to CSF (NfL-c) as diagnostic marker of MSA, and to assess NfL-p and NfL-c as markers of clinical disease progression.

**Methods:**

Well-characterized patients with early MSA (n=32), Parkinson’s disease (PD, n=21), and matched controls (CON, n=15) were enrolled in a prospective, longitudinal study of synucleinopathies with serial annual evaluations. NfL was measured using a high sensitivity immunoassay, and findings were assessed by disease category and relationship with clinical measures of disease progression.

**Results:**

Measurements of NfL-c were highly reproducible across immunoassay platforms (Pearson, r=0.99), while correlation between NfL-c and -p was only moderate (r=0.66). NfL was significantly higher in MSA compared to CON and PD; the separation was essentially perfect for NfL-c, but there was overlap, particularly with PD, for NfL-p. While clinical measures of disease severity progressively increased over time, NfL-c and -p remained at stable elevated levels within subjects across serial measurements. Neither change in NfL nor baseline NfL were significantly associated with changes in clinical markers of disease severity.

**Conclusions:**

These findings confirm NfL-c as faithful diagnostic marker of MSA, while NfL-p showed less robust diagnostic value. The significant NfL elevation in MSA was found to be remarkably stable over time and was not predictive of clinical disease progression.

## INTRODUCTION

Multiple system atrophy (MSA) is part of the “spectrum” of synucleinopathies, a group of neurodegenerative disorders that shares the pathophysiological mechanism of abnormal alpha-synuclein (αSyn) aggregation, which also includes Parkinson’s disease (PD) and dementia with Lewy-bodies (DLB).^1–3^ Clinically, MSA differs from PD and DLB by rapid disease progression and poor survival.^4–6^ MSA is also associated with more severe autonomic dysfunction and there is often cerebellar involvement.^3^ Misdiagnosis is nevertheless common, particularly at early disease stages or when the presentation is atypical.^7–9^

There are also important differences in pathology. The pathologic hallmark of MSA are glial cytoplasmic inclusions containing αSyn which differs from the Lewy-body pathology found in PD and DLB with inclusions predominantly within neurons.^10, 11^ More recently, we and others demonstrated differences in the αSyn structure between MSA and the Lewy-body synucleinopathies resulting in differences in protein misfolding assays which have been shown to allow for excellent diagnostic separation.^12–15^ However, these assays are highly complex and sensitive to even subtle assay variation, limiting their use currently to few specialized laboratories.^16^

We and others have also previously shown that neurofilament light chain (NfL) in spinal fluid (CSF, NfL-c) can serve as a diagnostic biomarker separating MSA from healthy controls (CON) and Lewy-body synucleinopathies.^15, 17, 18^ NfL-c can be measured using routine ELISA methodology which makes it an attractive, widely available biomarker. High values of NfL are not disease specific as they merely provide an index of central axonal degeneration and have therefore been reported in other neurologic conditions associated with rapid progression or considerable neuronal injury.^18, 19^ Nonetheless, when it comes to differentiating MSA from Lewy-body synucleinopathies, NfL-c can fulfill the unmet need for a reliable biomarker to solidify the clinical diagnosis.^15^

Since NfL crosses the blood-brain barrier in small amounts, there has been increasing interest in using NfL in plasma (NfL-p) as a more convenient biomarker for MSA and other neurologic conditions, though the low concentrations in plasma require special high-sensitivity assays.^19–21^ Furthermore, clinical tools, including the most widely accepted measure of disease severity, the Unified MSA Rating Scale (UMSARS), have recently been criticized for various limitations, underscoring the need for surrogate biomarkers of disease progression.^22–24^ Considering the use of NfL for that purpose in other neurologic conditions, it is logical to further explore such potential in MSA.^25–27^

In this prospective, longitudinal study, we sought to 1) confirm our previous findings of NfL in CSF as diagnostic marker for MSA utilizing prospectively collected CSF samples of clinically well-characterized and longitudinally followed patients with early MSA, PD, and healthy controls (CON) using a different, high sensitivity assay platform; 2) assess NfL in simultaneously collected plasma samples using the same platform, compare values in plasma with those in CSF, and assess the value of NfL-p as diagnostic marker for MSA; and 3) assess for change of NfL-c and -p over time with serial within subject samples, and explore if the change in NfL or baseline NfL are associated with or predict clinical disease progression starting with an early disease stage.

## METHODS

### Study Design

CSF and plasma samples were prospectively collected from well-characterized patients with early MSA, patients with PD, and matched CON subjects. Study subjects were followed longitudinally with annual collection of biomarkers as well as clinical measures of disease severity and progression for a duration of 3 years (4 evaluations).

### Participants

Subjects were enrolled as a part of a prospective, longitudinal study of synucleinopathies (Mayo Longitudinal Synucleinopathy Biomarker Study, NS092625).

Patients with MSA received a diagnosis of MSA-C or MSA-P by a Mayo Clinic movement disorder specialist. All patients had autonomic function testing to support the diagnosis. In order to be enrolled, patients were required to fulfill consensus criteria for possible or probable MSA and to have a score ≤17 (omitting the erectile dysfunction score) on part one of the Unified MSA Rating Scale (UMSARS) to ensure enrollment at an early disease stage.^28, 29^

Patients with PD were diagnosed by a Mayo Clinic movement disorder specialist and had to be at Hoehn and Yahr stage 2 to 3 in order to match the clinical severity of patients with early MSA. Healthy controls were without evidence of neurologic disease or autonomic dysfunction.

Subjects were excluded if they were pregnant or breastfeeding, scored 24 points or less on the Mini-Mental Status Examination, had a clinically significant or unstable medical or surgical condition that might preclude safe completion of the study or might affect the results of the study, or had taken any investigational products within 60 days prior to baseline.

### Standard Protocol Approvals, Registrations, and Patient Consents

The study was approved by the Mayo Clinic Institutional Review Board, and written informed consent was obtained from all participants.

### Clinical and Laboratory Study Assessments

A medical and neurologic history was obtained from all subjects and updated at the annual visits. All subjects underwent comprehensive general and neurologic examinations at each visit. These tasks, including all clinical scoring, were completed within Mayo’s outpatient Clinical Research and Trials Unit by the same investigator (WS) for all subjects and visits. Medications that could potentially bias evaluations were held for 5 half-lives prior to neurologic assessments, autonomic testing, blood draws, and spinal fluid collections. Neurologic impairment and deficits were quantified in MSA patients using the Unified MSA Rating Scale (UMSARS, consisting of part I which quantifies patients’ symptoms and function, part II which quantifies findings on neurologic examination, and part IV which comprises a global disability scale).^29^ The sum of UMSARS part I and part II is referred to as UMSARS Total. All subjects also underwent MRI of the head and autonomic function testing including autonomic reflex screen and thermoregulatory sweat testing annually. In order to quantify autonomic deficits, the Composite Autonomic Severity Score (CASS), a validated instrument to quantify the overall severity and distribution of autonomic failure based on standardized autonomic testing, was derived.^30^ Loss of thermoregulatory sweat function was quantified as percent anhidrosis (TST%).^31^ CASS was derived without as well as with consideration of TST findings (CASS-TST). Autonomic symptoms were assessed using the Composite Autonomic Severity Scale, COMPASS-select, including COMPASS-select subdomains of special interest (orthostatic, bladder).^32^

### CSF and Plasma Collection/Analysis

CSF and plasma were collected at each visit in conjunction with clinical, laboratory, and imaging assessments. Specimen collection took place between 3/1/2016 and 4/11/2022. Blood was collected in a fasting state via venipuncture into 10ml EDTA collection tubes, placed on ice, and immediately centrifuged at 3,500 rpm for 15 minutes at 4°C. The supernatant plasma was collected, aliquoted into cryovials, and stored at −80°C until the day of the bioassays.

CSF was collected via spinal tap. A lumbar spinal needle was placed in the subarachnoid space via posterior, intervertebral approach between lumbar level 2 and 5. After collection of CSF for safety assessments (cell count, protein), 10cc of CSF were collected into separate tubes for biomarker studies. CSF was placed on ice and processed immediately. After centrifuging at 10,000 rpm for 10 minutes at 4°C to remove any potential blood contamination, CSF was aliquoted into cryotubes and transferred to a −80° C freezer until the day of biomarker assays. Assays were performed with the technologist blinded to clinical information.

NfL was measured using an automated high-sensitivity immunoassay that allowed for quantitation of NfL at concentrations found in CSF as well as the much lower concentrations found in plasma with the same platform (Ella, ProteinSimple/Bio-Techne, Minneapolis, USA). In order to minimize measurement variability to the extent possible, all measurements were completed using same-lot microplates and longitudinal samples were allocated to the same microplate. For validation, measurements in CSF at baseline were compared to those previously completed and reported using conventional enzyme-linked immunosorbent assay (ELISA, Uman Diagnostics, Umea, Sweden) with an Omega Fluorescence Base microplate reader (BMG, Ortenberg, Germany).^15^ The Ella platform quantified NfL in triplicate for each sample and the average was used for further analysis and expressed in pg/ml.

### Statistical Analysis

Descriptive statistics were used to summarize demographic, clinical, and autonomic characteristics by disease group, including mean, standard deviation (SD), median, interquartile range (IQR), frequencies, and percentages as appropriate.

Given non-normal distributions in some groups, group differences in fluid markers, demographic, and clinical variables were compared using Kruskal-Wallis test for comparing multiple groups with pairwise post-hoc comparisons using Dunn test. Mann-Whitney U-test was used for a priori comparisons of only two groups. Receiver operating characteristic (ROC) curves and optimal cut-off values were derived for differentiating MSA samples from controls and PD.

Associations between fluid markers as well as between fluid markers and baseline characteristics were displayed visually and quantified using Pearson or Spearman correlation analysis as appropriate. Changes of variables over time were assessed using repeated measures ANOVA with post-hoc testing where appropriate. Mixed model analysis was used to explore if the baseline value or slope of change in NfL could predict progression of clinical measures. All statistical tests were 2-sided, and p values <0.05 were considered statistically significant. Analysis was performed using SPSS (IBM SPSS Statistics 28) and R version 4.2.2.

### Data Availability

The data that support the findings of this study are available from the corresponding author, upon reasonable request

## RESULTS

### Participants

A total of 68 subjects were included in this longitudinal study. The cohort consisted of 32 subjects with MSA (19 MSA-C), 21 PD, and 15 CON subjects. Demographic, clinical, and autonomic baseline characteristics are summarized in Table 1 by disease group. Patients with PD had longer disease duration and were slightly older than MSA and CON subjects. There was even gender distribution in the CON group, while there was male predominance in the MSA, and particularly in the PD group reflecting the known disease demographics of the synucleinopathies. Both autonomic deficits and autonomic symptom burden were notably higher in MSA patients compared to PD and CON subjects, as expected.

Twelve (38%) patients with a clinical diagnosis of MSA have come to autopsy, and all were confirmed as MSA. All other patients had a clinical disease course that was congruent with the clinical diagnosis based on longitudinal follow-up.

The median longitudinal follow-up duration with collection of biomarkers in this study was 2.3 years; while all three years of planned follow-up were accomplished in almost all CON subjects (93%), that percentage was lower in MSA (31%) and PD patients (43%) due to death, disease progression/disability, the COVID-19 pandemic, as well as patient drop-out because of enrollment into treatment trials.

### Cross-sectional NfL analysis - CSF

**Figure 1.**
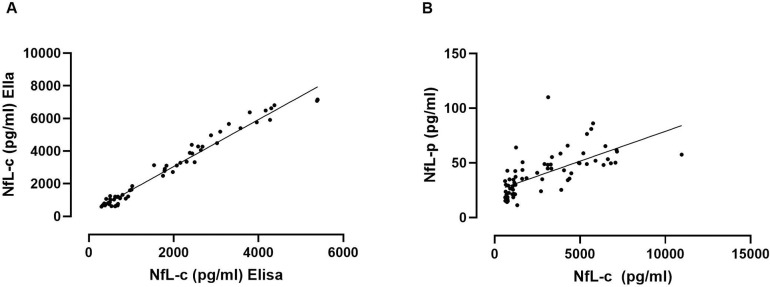
Neurofilament light chain correlation analyses. Correlation of NfL values in all subjects at baseline derived previously using conventional ELISA versus those derived using the high-sensitivity platform in this study (A). Correlation of baseline NfL in CSF versus NfL in plasma derived using the same high-sensitivity platform (B). There was remarkable agreement of values derived in CSF using different platforms, while the correlation between NfL in CSF and NfL in plasma was highly significant but of only moderate strength.

In order to validate measurements of NfL using the Ella platform against conventional ELISA methodology, we first assessed the degree of agreement between these methods. The baseline NfL-c data of most subjects in this study were originally measured using conventional ELISA as previously reported.^15^ We utilized these data to compare with measurements derived for the present study using the high-sensitivity Ella platform. There was remarkable agreement between both assays resulting in near-perfect correlation (Pearson, r=0.99, p<0.001, Figure 1A).

**Figure 2.**
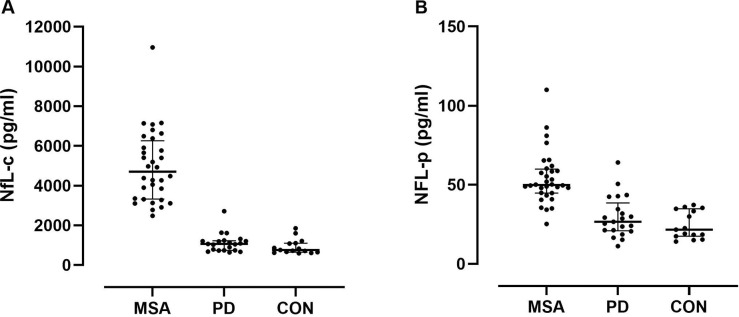
Comparison of neurofilament light chain between disease categories. NfL in MSA, PD, and CON subjects in CSF (A) and in plasma (B). There was perfect separation of MSA from CON subjects and near perfect separation from PD using NfL in CSF. There was also good separation of groups using NfL in plasma, but greater overlap with MSA, particularly with PD.

It is therefore not surprising that baseline NfL-c performed similarly well in this study separating early MSA from both CON and PD groups. NfL-c was markedly elevated in all MSA patients resulting in perfect separation from the CON group and near perfect separation from the PD group due to a single PD patient who had an NfL-c value entering the range of MSA patients (Figure 2A, both p<0.001, Dunn). There was no significant difference between CON and PD groups. There was no difference between MSA-P and MSA-C.

**Figure 3.**
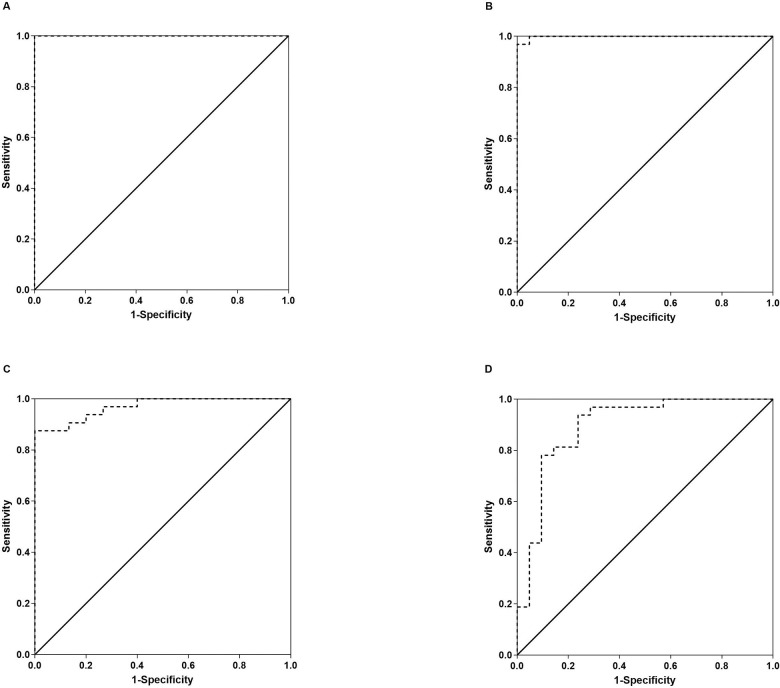
ROC curves. ROC curves for NFL-c differentiating MSA from CON subjects (A) and differentiating MSA from PD (B), as well as ROC curves for NfL-p differentiating MSA from CON (C) and from PD (D). NfL-c provided near perfect separation of MSA from the other groups, while the accuracy of separation was good but less robust for NfL-p.

For differentiating MSA from CON, we therefore found a perfect ROC curve for NfL-c with an area under the curve of 1.000 (Figure 3A). Any cut-off value between 1860 and 2484 pg/ml would result in 100% sensitivity and 100% specificity in differentiating MSA from CON. We also found a near perfect area under the curve (0.999) for the differentiation of MSA from PD (Figure 3B); while a cut-off value in the above cut-off range for CON would result in 100% sensitivity and 95% specificity in separating MSA from PD, the best cut-off value was found to be 2750 pg/ml which would result in 97% sensitivity but 100% specificity.

There was a significant association between NfL-c and age for CON (Pearson, r=0.71, p=0.003), but not for MSA or PD categories. No other significant demographic associations (sex, height, weight, BMI) were found. There were no significant associations between NfL-c and clinical measures of disease severity or disease duration in MSA.

### Cross-sectional NfL analysis - Plasma

In order to assess if levels of NfL in plasma reflect those in CSF, we compared measurements of NfL-c and NfL-p at baseline. Agreement was significant, but of only moderate strength (Pearson, r=0.66, p<0.001, Figure 1B). That was also the case when using data from all available timepoints.

Nevertheless, separation of MSA from CON and PD was highly significant in spite of overlap that was particularly prominent with the PD group (Figure 2B, p<0.001 and p=0.005, respectively, Dunn). ROC analysis for NfL-p differentiating MSA from CON revealed a respectable area under the curve of 0.969 (Figure 3C). A cut-off value of 39 pg/ml would provide 88% sensitivity and 100% specificity for differentiating MSA from CON. For the differentiation of MSA from PD, the ROC area under the curve was lower at 0.894 with a best cut-off value of 44 pg/ml achieving 78% sensitivity and 91% specificity in differentiating MSA from PD (Figure 3D).

There were no significant associations with demographic variables, including age, for NfL-p for either disease category after correction for multiple testing. There was no significant association between NfL-p and disease duration. There were weak associations between NfL-p and UMSARS I and IV which remained non-significant after correction for multiple testing.

### Longitudinal findings of clinical measures of disease severity, NfL, and their associations

**Figure 4.**
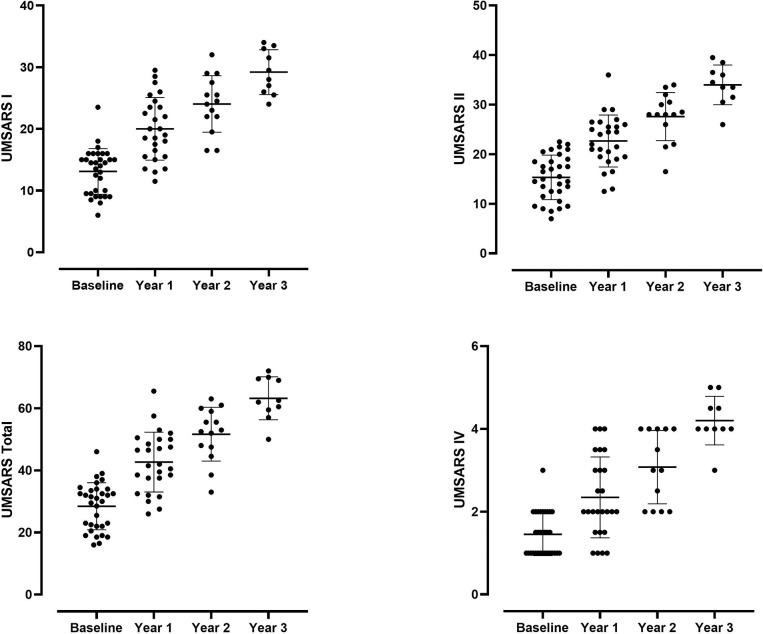
Progression of UMSARS scores in MSA patients over time. There was progressive and significant increase of all components of UMSARS (I, II, Total, and IV) over time.

Clinical measures of disease severity in MSA, including UMSARS I, UMSARS II, UMSARS Total, UMSARS IV, CASS, and CASS-TST increased progressively and significantly over time (UMSARS shown in Figure 4), except for TST% and COMPASS-select.

**Figure 5.**
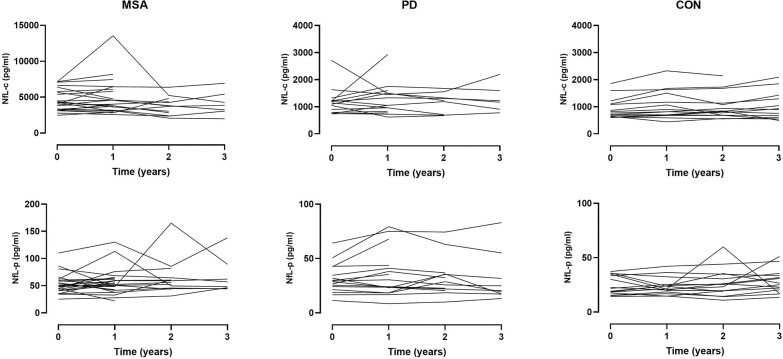
Longitudinal observations of neurofilament light chain. Spaghetti plots illustrating longitudinal change of NfL in CSF (top panels) and plasma (bottom panels) over serial measurements across disease categories. There was no significant change of NfL-c or NfL-p over time for either disease category, including MSA.

In contrast, there was no significant change of NfL-c or NfL-p over time for either disease category, including MSA (Figure 5). There was a greater degree of variability of NfL values observed in plasma compared to CSF.

It was therefore expected that change of NfL-c or NfL-p over time would not predict progression of clinical measures of disease severity, which was confirmed by mixed model analysis showing no significant NfL slope effect on the change of any of the clinical measures. Given that NfL is markedly elevated in MSA, we also tested if the baseline value of NfL in CSF or plasma would predict clinical disease progression. Mixed model analysis using NfL baseline values as independent and clinical disease severity measures over time as dependent variables did not reveal significant predictive value of baseline NfL for any clinical measure of disease progression with the exception of a very weak inverse relationship of baseline NfL-p and CASS (p=0.04, semi-partial R^2^ = 0.09).

## DISCUSSION

Misdiagnosis of MSA remains a common problem and when the diagnosis is made correctly, the disease is often clinically quite advanced.^4–9^ Whether or not the recently revised diagnostic criteria can improve this situation is yet to be determined.^33^ On the other hand, diagnostic accuracy is an important attribute of clinical care of patients who face a terminal diagnosis.

Efforts towards disease-modifying therapies in MSA have seen a remarkable surge in recent years. Current trials have set inclusion criteria that not only aim to ensure a correct diagnosis, but also limited functional impairment. The rationale for the latter likely reflect efforts to capture a still responsive disease stage, but also concerns about a ceiling effect of clinical outcome measures.^23, 24, 34^ This situation underlines the need for enhancing diagnostic certainty at an early disease stage.

We and others have recently described CSF biomarkers with remarkable diagnostic potential in differentiating MSA from other synucleinopathies, including PMCA/RT-QuIC and NfL.^12–15^ The protein misfolding assays mimic in vitro the seeding potential of misfolded aSyn resulting in progressive aggregation of native aSyn that is provided as substrate; the resulting aggregates differ between MSA and the Lewy-body synucleinopathies and allow for their faithful differentiation.^14^ NfL on the other hand represents a marker of axonal degeneration that has been demonstrated to be elevated in a number of neurodegenerative, neuroinflammatory, and neurotraumatic conditions associated with rapid progression, high disease activity, and/or severe axonal damage.^19, 35, 36^ In neurodegenerative disorders, NfL likely reflects the tempo and distribution of neuronal degeneration. While elevated levels of NfL are therefore not disease-specific, NfL in CSF has been shown to provide very accurate differentiation between MSA and the Lewy-body synucleinopathies, even before motor symptoms develop.^15, 17, 18, 37^ In contrast to the highly complex and time-consuming protein aggregation assays, measurements of NfL can be readily achieved using ELISA technology.^16^

Since a small amount of NfL crosses the blood-brain barrier, it comes as no surprise that NfL has also been shown to be elevated in plasma in disorders associated with elevated NfL in the central nervous system.^19, 36^ Since NfL in plasma occurs in much lower concentrations compared to CSF, measurements of NfL-p require more specialized high sensitivity assay platforms, which have recently become more readily available. Even though one would expect the much lower concentrations of NfL in plasma to be more variable and - given both central and peripheral sources - less specific, the prospect of a blood-based biomarker is certainly nevertheless highly intriguing.^19^

Measuring disease progression in MSA remains challenging. Although a validated, disease-specific, and widely accepted measure of clinical disease severity exists, the UMSARS has received increasing criticism.^22, 24^ Specific areas of concern include limitation of responsiveness to progression, ceiling effect, content and construct validity, and reliability. A taskforce was recently formed to address these concerns and revise the current instrument^23^ In light of trials of disease-modification which uniformly utilize UMSARS as primary outcome measure, it is a major concern that the current limitations result in significant variability exceeding the expected effect size of candidate agents.^23^ A biomarker-based surrogate marker of disease progression would therefore be highly valuable not only for prognostic purposes, but also for measuring target-engagement of disease-modifying therapies. Since 1) there are conflicting reports and opinions about increase of NfL with disease progression in MSA, 2) there are reports that NfL may predict clinical progression, 3) NfL has been reported as biomarker of disease activity and treatment response in other neurologic disorders, and 4) prior work on this topic is limited to NfL in plasma, further systematic exploration of the role of NfL as marker of disease progression in MSA using serial longitudinal biomarkers in plasma and CSF along with clinical assessments in a carefully phenotyped cohort seemed prudent.^20, 21, 38, 39^

This study was therefore designed to 1) confirm our findings of NfL in CSF as diagnostic marker of early MSA using a different, high-sensitivity platform; 2) assess NfL in the plasma of the same patients using the same assay platform, compare values in plasma with those in CSF, and assess NfL-p as diagnostic marker; and 3) assess for change of NfL in both CSF and plasma over time starting at an early disease stage, and explore if the change in NfL or NfL at baseline are associated with or predict clinical disease progression.

We established that measuring NfL is remarkably reproducible across different immunoassay platforms showing perfect agreement. Moreover, we found again clean separation of MSA patients not only from CON subjects, but also from patients with PD, who were studied at a stage of comparable disease severity. NfL-c above 2000 pg/ml allowed for perfect separation of MSA patients from CON subjects, while values above 2750 pg/ml provided near-perfect separation from PD (97% sensitivity, 100% specificity) which was as good if not better as previously reported.^15, 17–19^

On the other hand, we found only moderate correlation between NfL-c and NfL-p consistent with previous reports for neurodegenerative disorders.^20, 26^ The probably most compelling explanation for this lies in the fact that axonal degeneration in MSA is predominantly a central process and NfL elevation in plasma therefore mostly the result of spill-over across the blood-brain barrier. Since that barrier is furthermore known to be more “leaky” in MSA, another element of variability is added.^40^ On the other hand, NfL-p is also known to be elevated in peripheral neuropathies indicating a peripheral contribution to plasma NfL.^41, 42^ One may therefore expect to see transient elevations as a result of falls and other injuries resulting in soft tissue damage, which are common in MSA, particularly since the levels of NfL in plasma are very low. This may well add to the higher variability of NfL-p we observed over time compared to NfL-c.

NfL-p nevertheless provided excellent separation between MSA and CON subjects and still respectable separation from PD, yet in contrast to NfL-c, there was overlap. NfL-p above 39 pg/ml had 88% sensitivity and 100% specificity for separating MSA from CON, while values above 44 pg/ml provided separation of MSA from PD with 78% sensitivity and 91% specificity. These findings are consistent with a previous report exploring NfL-p in PD versus atypical parkinsonian disorders.^43^ One could argue that this differentiation provided by a blood-based marker is potentially useful as a supportive diagnostic marker. However, we would argue that when it comes to the diagnosis of a fatal neurodegenerative disease, the notably higher accuracy of NfL-c justifies obtaining spinal fluid.

Since previous reports on longitudinal change of NfL-c in MSA are lacking and recent reports on NfL-p indicated a mild but significant increase over time, our longitudinal findings based on annual CSF and plasma collections were somewhat unexpected. While NfL was markedly elevated, this elevation remained stable over time with serial measurements in both CSF and plasma, in spite of significant progression of clinical markers. This stability was remarkably uniform across patients and showed little fluctuation, particularly for NfL-c. This would be congruent with the conclusion that NfL is more reflective of disease activity than disease burden, which may be a stable intraindividual disease attribute. Alternatively, it may reflect a relatively stable balance between disease burden and diminishing neuronal pool. There are important differences between our study and two other recent longitudinal reports suggesting an increase of NfL-p over time in that our patients underwent serial NfL measurements annually instead of just two measurements with variable collection interval, measurements were not only performed in plasma but also CSF with the same longitudinal trend observed in both, and we ensured enrollment at a homogenous, early disease stage reflecting inclusion criteria of disease-modifying trials, which may explain differences in findings.^20, 21^ We also did not see significant associations of NfL with disease duration or markers of disease severity, although there was a weak trend of an association for two clinical severity markers that was only seen for NfL-p but not for NfL-c. Since other recent reports indicate a weak but significant association of NfL-p with disease severity, a possible explanation for this finding in plasma but not spinal fluid may be greater contribution to NfL elevations from falls and injuries or greater impairment of the blood-brain-barrier as the disease progresses; such association would be expected to be more evident in studies without focus on early disease.^20, 21, 40^

Given the lack of increase of NfL over time, we did not expect to find associations between changes of NfL and progression of clinical markers, which was confirmed with dedicated mixed model analyses. We also did not find an association of high NfL with more rapid disease progression, neither for NfL-c nor NfL-p. Although there are no prior reports of such association in CSF, two recent studies reported weak associations between plasma NfL and disease progression; it is therefore possible that a larger sample size may have revealed a weak association, but even if that was the case we would anticipate such association to not be clinically useful as progression marker.^20, 21^ A more likely explanation may again be the much wider range of disease severity enrolled in the other trials, which therefore may have seen more NfL-p elevations as a result of falls and injuries. One might also speculate that the known limitations of available clinical measures of disease severity may be playing a role, though most of the clinical measures demonstrated consistent disease progression. It is furthermore possible that the global severity of central axonal degeneration in MSA does not closely reflect the speed and magnitude of axonal loss in brain regions critical for loss of functional abilities and changes on neurologic examination. In order to better understand this conundrum, it is going to be important to explore the trajectory of NfL in even earlier, prodromal disease stages on the one hand, and on the other hand to study the effects of disease-modifying therapy candidates in MSA not only on clinical disease progression, but also on NfL as well as quantitative MRI measures, as such multi-dimensional assessment in aggregate may help explain the apparently complex relationship between clinical disease progression and measures of axonal degeneration. It would be expected that an effective drug candidate would result in a decrease of the otherwise stable intra-individual NfL levels as has been shown in other neurologic conditions. In that case, NfL would have a role as measure of intraindividual disease activity.

The strengths of our study include 1) inclusion of patients specifically at an early disease stage since a) at that stage diagnostic biomarkers would have the greatest clinical utility, b) that stage is congruent with the stage aimed for in clinical trials, and c) this stage reflects the area of greatest need for markers of disease progression; 2) the prospective design, serial longitudinal follow-up, and longitudinal biomarker collection over several years, which helped ascertain the correct diagnosis of all patients enrolled and allowed for assessment of change of both clinical disease progression and biomarkers; 3) collection of both plasma and CSF at all serial visits which allowed for cross-sectional and longitudinal measurements and comparisons of NfL in both body fluids; 4) meticulous and longitudinal clinical, autonomic, and imaging phenotyping of all subjects; 5) the standardized, rigorously careful handling and analysis of samples in this single center study. Weaknesses include differences in gender distribution and mild differences in age between the groups as well as the drop-out of patients over time resulting in smaller patient numbers after the first one to two years, which, however, does reflect the devastating nature of the disease under study.

In conclusion, our findings confirm the remarkable diagnostic value of NfL in CSF which confidently distinguishes early MSA from healthy controls as well as PD across different analysis platforms. We demonstrate only moderate correlation between CSF and plasma values of NfL, but on the other hand reasonable separation of MSA from PD using plasma NfL which could serve as a “poor man’s” marker of MSA when CSF collection is either not feasible or contraindicated. Our data furthermore demonstrate lack of longitudinal change of NfL in MSA, in fact remarkable stability of NfL values over several years. NfL was furthermore not associated with clinical markers of disease progression. Therefore, while NfL, particularly in CSF, is confirmed here as valuable diagnostic marker, it does not possess properties that would support a role as marker of clinical disease severity or progression. However, it may nevertheless reflect target engagement in clinical trials of disease modification if a convincing decline in NfL values could be demonstrated.

## Figures and Tables

**Table 1.** Demographic, clinical, and autonomic characteristics by disease group. Normally distributed numeric variables are shown as mean and standard deviation, not normally distributed variables as median and IQR. Frequencies are reported as number and category percentage.

Category	MSA	CON	PD
**Number**	32	15	21
**Age, years**	61.1±6.8	61.7±6.0	66.0±6.7
**Sex (%) male**	22 (69)	7 (47)	18 (86)
**Disease duration, years**	3.6 (IQR 2.6–55)	−	7.3 (IQR 5.3–10.9)
**MSA-C (%)**	19 (59)	−	−
**UMSARS I**	13.1±3.7	−	−
**UMSARS II**	15.3±4.5	−	−
**UMSARS Total**	28.4±7.6	−	−
**COMPASS-select**	31.9±17.9	2.4±2.3	19.5±16.9
**CASS**	4.1±2.0	0.5±0.9	3.0±2.9
**TST (% anhidrosis)**	75.7 (IQR 17.2–90.9)	2.1 (IQR 0.4–19.9)	3.2 (IQR 1.7–45.2)

## References

[1] Boeve BF, Silber MH, Ferman TJ, Association of REM sleep behavior disorder and neurodegenerative disease may reflect an underlying synucleinopathy. Mov Disord 2001;16:622–630. 11481685 10.1002/mds.1120

[2] Coon EA, Singer W. Synucleinopathies. Continuum (Minneap Minn) 2020;26(1):72–92. 31996623 10.1212/CON.0000000000000819 PMC7745651

[3] Stankovic I, Fanciulli A, Sidoroff V, Wenning GK. A Review on the Clinical Diagnosis of Multiple System Atrophy. Cerebellum 2022; Aug 19. 10.1007/s12311-022-01453-w PMC10485100 35986227

[4] Coon EA, Suarez MD, Ahlskog JE, Survival in Multiple System Atrophy – Insights from a Large Retrospective Cohort. Ann Neurol 2014;76:S39 (abstract).

[5] Low PA, Reich SG, Jankovic J, Natural history of multiple system atrophy in the USA: a prospective cohort study. Lancet Neurol 2015;14:710–719. 26025783 10.1016/S1474-4422(15)00058-7 PMC4472464

[6] Wenning GK, Geser F, Krismer F, The natural history of multiple system atrophy: a prospective European cohort study. Lancet Neurol 2013;12:264–274. 23391524 10.1016/S1474-4422(12)70327-7 PMC3581815

[7] Hughes AJ, Daniel SE, Ben-Shlomo Y, Lees AJ. The accuracy of diagnosis of parkinsonian syndromes in a specialist movement disorder service. Brain 2002;125(Pt 4):861–870. 11912118 10.1093/brain/awf080

[8] Koga S, Aoki N, Uitti RJ, When DLB, PD, and PSP masquerade as MSA: an autopsy study of 134 patients. Neurology 2015;85:404–412. 26138942 10.1212/WNL.0000000000001807 PMC4534078

[9] Osaki Y, Wenning GK, Daniel SE, Do published criteria improve clinical diagnostic accuracy in multiple system atrophy? Neurology 2002;59:1486–1491. 12455559 10.1212/01.wnl.0000028690.15001.00

[10] Papp MI, Kahn JE, Lantos PL. Glial cytoplasmic inclusions in the CNS of patients with multiple system atrophy (striatonigral degeneration, olivopontocerebellar atrophy and Shy-Drager syndrome). J Neurol Sci 1989;94:79–100. 2559165 10.1016/0022-510x(89)90219-0

[11] Trojanowski JQ, Revesz T. Proposed neuropathological criteria for the post mortem diagnosis of multiple system atrophy. Neuropathol Appl Neurobiol 2007;33:615–620. 17990994 10.1111/j.1365-2990.2007.00907.x

[12] Poggiolini I, Gupta V, Lawton M, Diagnostic value of cerebrospinal fluid alpha-synuclein seed quantification in synucleinopathies. Brain 2022;145:584–595. 34894214 10.1093/brain/awab431 PMC9014737

[13] Rossi M, Candelise N, Baiardi S, Ultrasensitive RT-QuIC assay with high sensitivity and specificity for Lewy body-associated synucleinopathies. Acta Neuropathol 2020;140:49–62. 32342188 10.1007/s00401-020-02160-8 PMC7299922

[14] Shahnawaz M, Mukherjee A, Pritzkow S, Discriminating alpha-synuclein strains in Parkinson’s disease and multiple system atrophy. Nature 2020;578:273–277. 32025029 10.1038/s41586-020-1984-7 PMC7066875

[15] Singer W, Schmeichel AM, Shahnawaz M, Alpha-Synuclein Oligomers and Neurofilament Light Chain in Spinal Fluid Differentiate Multiple System Atrophy from Lewy Body Synucleinopathies. Ann Neurol 2020;88:503–512. 32557811 10.1002/ana.25824 PMC7719613

[16] Martinez-Valbuena I, Visanji NP, Kim A, Alpha-synuclein seeding shows a wide heterogeneity in multiple system atrophy. Transl Neurodegener 2022;11:7. 35125105 10.1186/s40035-022-00283-4 PMC8819887

[17] Abdo WF, Bloem BR, Van Geel WJ, CSF neurofilament light chain and tau differentiate multiple system atrophy from Parkinson’s disease. Neurobiol Aging 2007;28:742–747. 16678934 10.1016/j.neurobiolaging.2006.03.010

[18] Hall S, Ohrfelt A, Constantinescu R, Accuracy of a panel of 5 cerebrospinal fluid biomarkers in the differential diagnosis of patients with dementia and/or parkinsonian disorders. Arch Neurol 2012;69:1445–1452. 22925882 10.1001/archneurol.2012.1654

[19] Wang SY, Chen W, Xu W, Neurofilament Light Chain in Cerebrospinal Fluid and Blood as a Biomarker for Neurodegenerative Diseases: A Systematic Review and Meta-Analysis. J Alzheimers Dis 2019;72:1353–1361. 31744001 10.3233/JAD-190615

[20] Chelban V, Nikram E, Perez-Soriano A, Neurofilament light levels predict clinical progression and death in multiple system atrophy. Brain 2022;145:4398–4408. 35903017 10.1093/brain/awac253 PMC9762941

[21] Zhang L, Cao B, Hou Y, Neurofilament Light Chain Predicts Disease Severity and Progression in Multiple System Atrophy. Mov Disord 2022;37:421–426. 34719813 10.1002/mds.28847

[22] Foubert-Samier A, Pavy-Le Traon A, Saulnier T, An Item Response Theory analysis of the Unified Multiple System Atrophy Rating Scale. Parkinsonism Relat Disord 2022;94:40–44. 34875563 10.1016/j.parkreldis.2021.11.024

[23] Krismer F, Palma JA, Calandra-Buonaura G, The Unified Multiple System Atrophy Rating Scale: Status, Critique, and Recommendations. Mov Disord 2022;37:2336–2341. 36074648 10.1002/mds.29215 PMC9771866

[24] Palma JA, Vernetti PM, Perez MA, Limitations of the Unified Multiple System Atrophy Rating Scale as outcome measure for clinical trials and a roadmap for improvement. Clin Auton Res 2021;31:157–164. 33554315 10.1007/s10286-021-00782-w PMC7868077

[25] Dreger M, Steinbach R, Gaur N, Cerebrospinal Fluid Neurofilament Light Chain (NfL) Predicts Disease Aggressiveness in Amyotrophic Lateral Sclerosis: An Application of the D50 Disease Progression Model. Front Neurosci 2021;15:651651. 33889072 10.3389/fnins.2021.651651 PMC8056017

[26] Mattsson N, Cullen NC, Andreasson U, Association Between Longitudinal Plasma Neurofilament Light and Neurodegeneration in Patients With Alzheimer Disease. JAMA Neurol 2019;76:791–799. 31009028 10.1001/jamaneurol.2019.0765 PMC6583067

[27] Rojas JC, Wang P, Staffaroni AM, Plasma Neurofilament Light for Prediction of Disease Progression in Familial Frontotemporal Lobar Degeneration. Neurology 2021;96:e2296–e2312. 33827960 10.1212/WNL.0000000000011848 PMC8166434

[28] Gilman S, Wenning GK, Low PA, Second consensus conference on the diagnosis of multiple system atrophy. Neurology 2008;71:670–676. 18725592 10.1212/01.wnl.0000324625.00404.15 PMC2676993

[29] Wenning GK, Tison F, Seppi K, Development and validation of the Unified Multiple System Atrophy Rating Scale (UMSARS). Mov Disord 2004;19:1391–1402. 15452868 10.1002/mds.20255

[30] Low PA. Composite autonomic scoring scale for laboratory quantification of generalized autonomic failure. Mayo Clin Proc 1993;68:748–752. 8392653 10.1016/s0025-6196(12)60631-4

[31] Fealey RD, Low PA, Thomas JE. Thermoregulatory sweating abnormalities in diabetes mellitus. Mayo Clinic Proc 1989;64:617–628. 10.1016/s0025-6196(12)65338-5 2747292

[32] Lipp A, Sandroni P, Ahlskog JE, Prospective differentiation of multiple system atrophy from Parkinson disease, with and without autonomic failure. Arch Neurol 2009;66:742–750. 19506134 10.1001/archneurol.2009.71 PMC2838493

[33] Wenning GK, Stankovic I, Vignatelli L, The Movement Disorder Society Criteria for the Diagnosis of Multiple System Atrophy. Mov Disord 2022;37:1131–1148. 35445419 10.1002/mds.29005 PMC9321158

[34] Singer W, Low PA. Optimizing clinical trial design for multiple system atrophy: lessons from the rifampicin study. Clin Auton Res 2015;25:47–52. 25763826 10.1007/s10286-015-0281-2 PMC4763681

[35] Bagnato S, Grimaldi LME, Di Raimondo G, Prolonged Cerebrospinal Fluid Neurofilament Light Chain Increase in Patients with Post-Traumatic Disorders of Consciousness. J Neurotrauma 2017;34:2475–2479. 28385104 10.1089/neu.2016.4837

[36] Varhaug KN, Torkildsen O, Myhr KM, Vedeler CA. Neurofilament Light Chain as a Biomarker in Multiple Sclerosis. Front Neurol 2019;10:338. 31024432 10.3389/fneur.2019.00338 PMC6460359

[37] Singer W, Schmeichel AM, Shahnawaz M, Alpha-Synuclein Oligomers and Neurofilament Light Chain Predict Phenoconversion of Pure Autonomic Failure. Ann Neurol 2021;89:1212–1220. 33881777 10.1002/ana.26089 PMC8168720

[38] Kuhle J, Plavina T, Barro C, Neurofilament light levels are associated with long-term outcomes in multiple sclerosis. Mult Scler 2020;26:1691–1699. 31680621 10.1177/1352458519885613 PMC7604552

[39] Palleis C, Morenas-Rodriguez E, Murcia FJM, Longitudinal correlation between neurofilament light chain and UMSARS in Multiple System Atrophy. Clin Neurol Neurosurg 2020;195:105924. 32512475 10.1016/j.clineuro.2020.105924

[40] Song SK, Lee SK, Lee JJ, Blood-brain barrier impairment is functionally correlated with clinical severity in patients of multiple system atrophy. Neurobiol Aging 2011;32:2183–2189. 20149484 10.1016/j.neurobiolaging.2009.12.017

[41] Amaador K, Wieske L, Koel-Simmelink MJA, Serum neurofilament light chain, contactin-1 and complement activation in anti-MAG IgM paraprotein-related peripheral neuropathy. J Neurol 2022;269:3700–3705. 35157138 10.1007/s00415-022-10993-4 PMC9217848

[42] Huehnchen P, Schinke C, Bangemann N, Neurofilament proteins as a potential biomarker in chemotherapy-induced polyneuropathy. JCI Insight 2022;7: e154395. 35133982 10.1172/jci.insight.154395 PMC8986065

[43] Hansson O, Janelidze S, Hall S, Blood-based NfL: A biomarker for differential diagnosis of parkinsonian disorder. Neurology 2017;88:930–937. 28179466 10.1212/WNL.0000000000003680 PMC5333515

